# Supplementary material for: A case report describing the immune response of an infant with congenital heart disease and severe COVID-19
Source: Commun Med (Lond). 2021 Nov 15;1:47. doi: 10.1038/s43856-021-00047-7 (PMC9053208; doi:10.1038/s43856-021-00047-7)
Supplement: Supplementary file 1 — Supplementary Information [file 43856_2021_47_MOESM1_ESM.pdf]

Supplementary Information.

Supplementary Table 1: Baseline laboratory investigations in an infant with severe COVID-19.

Microbiological	Haematological and biochemical	Inflammatory markers
• SARS-CoV-2 PCR: positive (oro/nasopharyngeal specimens and urine; equivocal in blood)<br><br>• Blood cultures: negative	• Haemoglobin: 136 g/L (NR 95 - 135)<br><br>• Platelets: **79** x10e9/L (NR 150 - 400)<br><br>• Total white cell count: **3.9** x10e9/L (NR 6.0 - 18.0)<br><br>• Lymphocytes: **0.43**x10e9/L (NR 4.0 - 10.0)<br><br>• Neutrophils: 3.24 (NR 1 - 8.5x10e9/L)<br><br>• Urea: 3.7 mmol/L (NR 1.3 - 6.6)<br><br>• Creatinine: 17 µmol/L (NR 10 - 30)<br><br>• Alanine aminotransferase: **133** IU/L (NR <50)	• Ferritin: **9487 µg/L** (NR 11-87)<br><br>• Lactate dehydrogenase: **2060 U/L** (NR 120-246)<br><br>• Procalcitonin: **16 µg/L** (NR <0.06)<br><br>• D-dimer: **5.86 µg/mL** (NR<0.5)<br><br>• Troponin: **0.7 ng/mL** (NR <0.034)

NR=normal range

Supplementary Table 2: Viral load inferred from cycle threshold (Ct) values from nasopharyngeal/oropharyngeal (NOP) and urine samples demonstrating rapid reduction in viral load over the course of the illness.

Time-point (in relation to admission to ICU/intubation, T=0)	Date of collection	Cycle threshold (Ct value)	Quantification cycle (Cq) and viral load (copies/ml) in urine samples		
			RdRp[#]	E[^]	RdRp[^]
Day -2 (symptom onset)	12/6	8.81*			
Day 3	17/6	18.45	32.877<br>59375.582	33.692<br>9464.104	37.03<br>5858.09
Day 5	19/6	25.8	Not detected	Not detected	Not detected
Day 6	20/6	28.57	-	-	-
Day 10	24/6	33.47	Not detected	Not detected	Not detected
Day 15	29/6	32.88	-	-	-
Day 22	6/7	33.6	-	-	-
Day 28	12/7	Not detected	Not detected	Not detected	Not detected
Day 107	29/9	Not detected	Not detected	Not detected	Not detected

*Cycle threshold values from the TibMolbiol LightMix Modular SARS-CoV(COVID19) E-gene for day 5-28 samples. *Day -2 sample performed at symptom onset, at a different hospital laboratory using AusDiagnostics High-Plex system utilising Multiplex Tandem PCR (MT-PCR). References for # and ^ are added at the end of this document.*

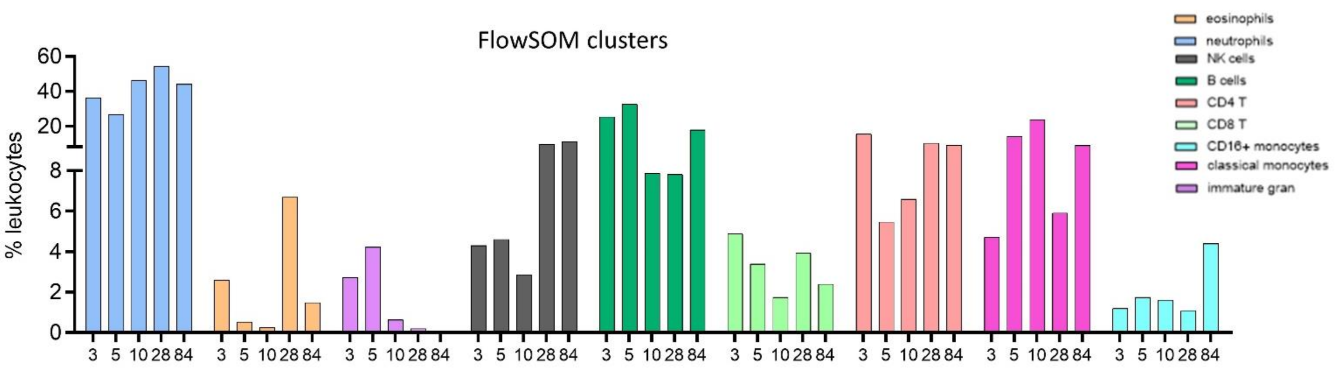

Supplementary Figure 1. Unsupervised analysis of whole blood flow cytometry data. Annotated FlowSOM cluster frequencies for neutrophils, eosinophils, immature granulocytes, NK cells, B cells, CD8 T cells, CD4 T cells, classical monocytes and CD16+ monocytes as proportion of leukocytes (CD45+ live single cells). FlowSOM = Flow Self Organising Maps

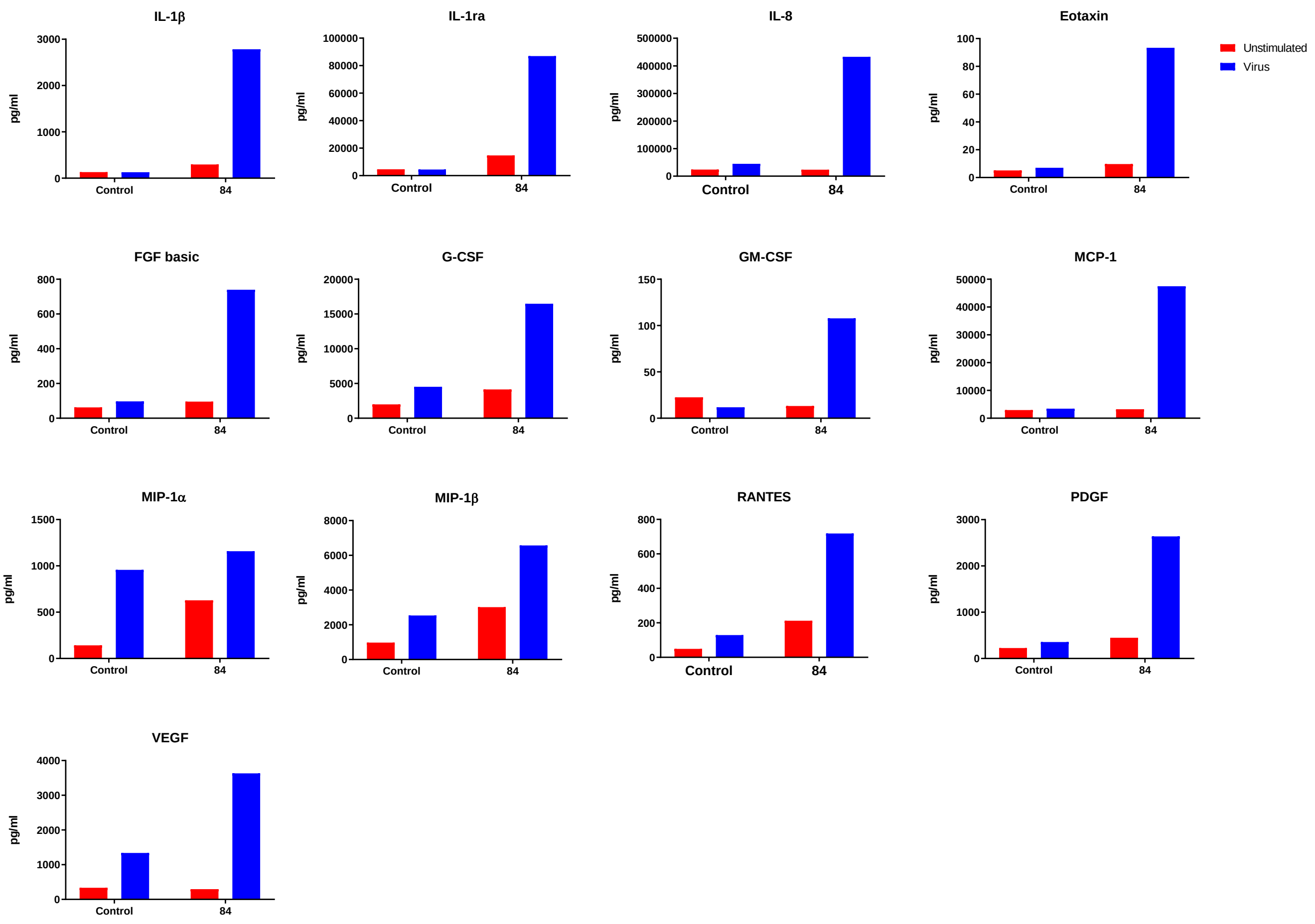

Supplementary Figure 2: Additional cytokines/chemokines upregulated following SARS-CoV-2 stimulation of peripheral blood mononuclear cells at Day 84. Following stimulation for 4 days we observed upregulation of IL-1β, IL-1ra, IL-8, eotaxin, FGF basic, G-CSF, GM-CSF, MCP-1, MIP-1α, MIP-1β, RANTES, PDGF and VEGF in the SARS-CoV-2 infected patient but not in the control.

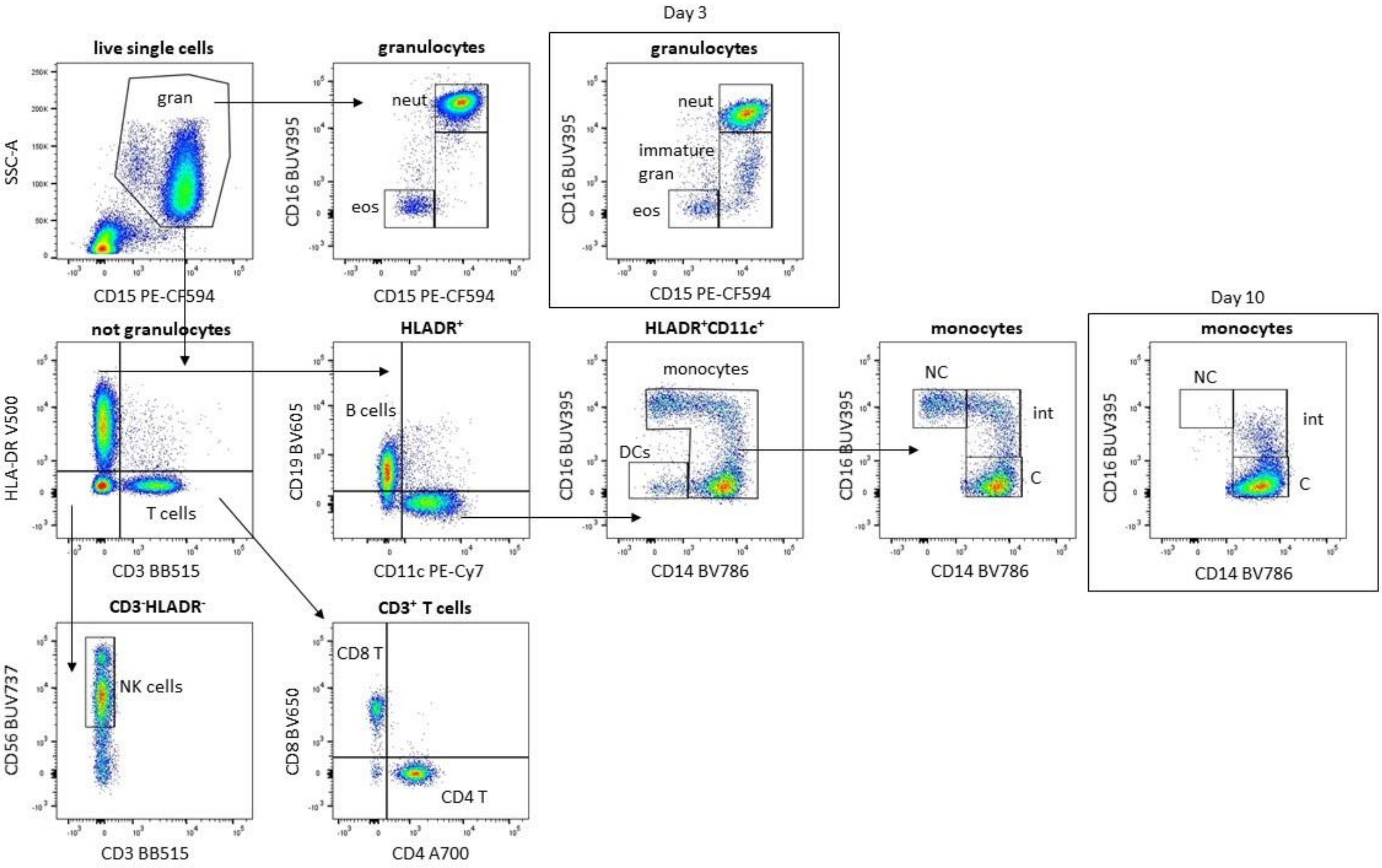

Supplemental Figure 3: Whole blood flow cytometry gating strategy. Granulocytes were selected within $CD45^{+}$ leukocytes based on their SSC profile and CD15 expression. Neutrophils were $CD15^{+}CD16^{+}$ and eosinophils were $CD15^{+}CD16^{-}$. Intermediate granulocytes were identified during the first 10 days, represented here at day 3. Within the non-granulocyte fraction, CD3 T cells were identified and classified into CD4 and CD8 T cells. B cells were identified based on CD19 and HLA-DR expression. $CD11c^{+}CD14^{+}$ monocytes and their subsets were gated based on CD16 expression (NC: non classical, int: intermediate and C: classical). Total dendritic cells were $HLADR^{+}CD11c^{+}CD14^{-}$ and NK cells were $HLADR^{-}CD3^{-}CD56^{+}$ cells

Supplementary Table 3: Flow cytometry antibody cocktail for whole blood phenotyping

Surface Marker	Fluorophore	Clone	Final Dilution
CD14	BV786	M5E2	1:50
CD11b	BUV805	ICRF44	1:100
CD45	BV711	HI30	1:100
CD56	BUV737	NCAM16.2	1:100
CD11c	PE-Cy7	B-ly6	1:100
CD63	A647	H5C6	1:100
CD4	A700	RPA-T4	1:100
CD3	BB515	VCHTI	1:100
CD15	PE-CF594	W6D3	1:200
HLADR	V500	G46-6	1:200
CD19	BV605	5J25C1	1:200
CD8	BV650	RPA-T8	1:200
CD16	BUV395	3G8	1:400
Live/dead	N-IR		

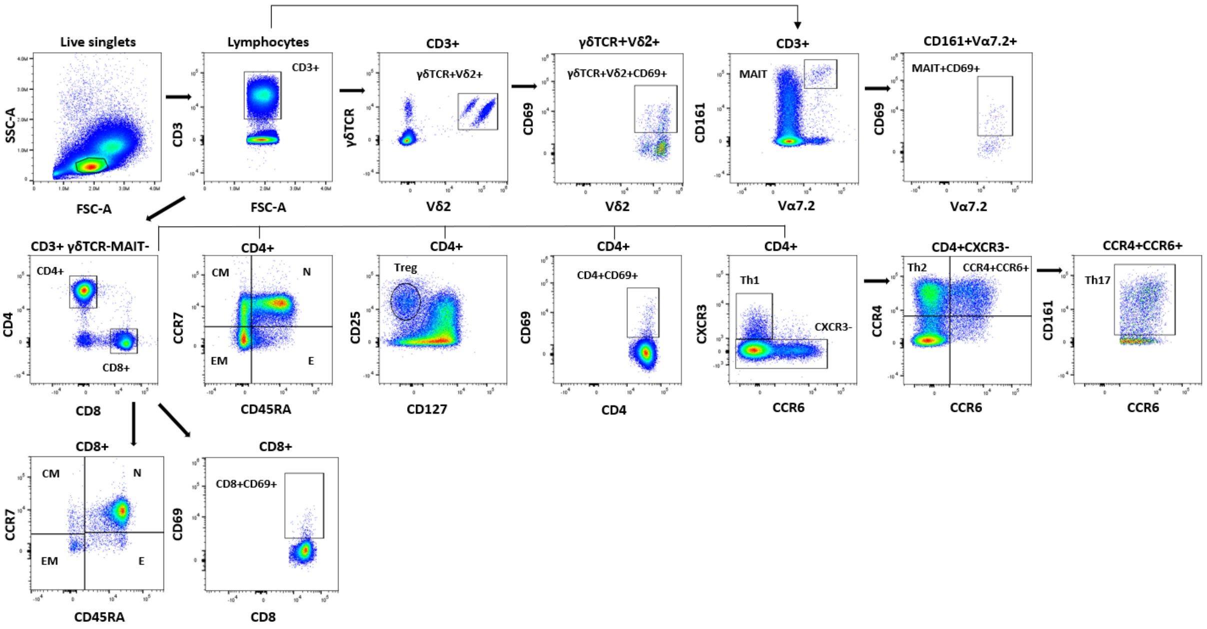

Supplementary Figure 4. Peripheral blood mononuclear cells gating strategy: T-cells were identified by CD3+ expression on live single lymphocytes. T-cells were further categorised into CD4+, CD8+, γδTCR+Vδ2+ and CD161+Vα7.2+ (MAIT cells). γδTCR+ cells and MAIT cells were gated out from CD4+ and CD8+ T-cells. From the CD4+ T-cells we further identified their subsets. CXCR3+ cells were considered Th1, CXCR3-CCR4+CCR6- cells were considered Th2, CXCR3-CCR4+CCR6+CD161+ cells were considered Th17 and $CD25^{hi}CD127^{lo}$ cells were considered Tregs. CCR7 and CD45RA were used to discriminate CD4+ and CD8+ cells into their memory subsets (Central memory (CM), effector memory (EM), naïve (N) and effector (E)). To determine activation status, CD69 was gated from all T-cell subsets, CD4+, CD8+, γδTCR+Vδ2+ and MAIT cells.

Supplementary Table 4: Flow cytometry antibody cocktail for peripheral blood mononuclear cell phenotyping

Surface Marker	Fluorophore	Clone	Final Dilution
CD3	BUV395	UCHT1	1:100
CD4	BV510	SK3	1:200
CD8	BUV805	SK1	1:400
CD25	PE-CF594	M-A251	1:100
CD45RA	PerCP/Cy5.5	MI100	1:200
CD69	BV650	FN50	1:200
CD127	APCR700	HIL-7R-M21	1:100
CD161	PEvio770	191B8	1:200
CXCR3	APC	IC6/CXCR3	1:50
CCR4	BV605	L29IH4	1:200
CCR6	BV421	11A9	1:100
CCR7	BV785	G043H7	1:100
γδTCR	FITC	11F2	1:50
Vδ2	PE	B6	1:400
Vα7.2	BV711	3C10	1:200
Zombie NIR	NIR		1:800

Supplementary References

Chan, J. F. et al. Improved Molecular Diagnosis of COVID-19 by the Novel, Highly Sensitive and Specific COVID-19-RdRp/Hel Real-Time Reverse Transcription-PCR Assay Validated In Vitro and with Clinical Specimens. Journal of clinical microbiology 58, doi:10.1128/JCM.00310-20 (2020).[11]

^ Corman VM, Landt O, Kaiser M, Molenkamp R, Meijer A, Chu DK, Bleicker T, Brünink S, Schneider J, Schmidt ML, Mulders DG, Haagmans BL, van der Veer B, van den Brink S, Wijsman L, Goderski G, Romette JL, Ellis J, Zambon M, Peiris M, Goossens H, Reusken C, Koopmans MP, Drosten C. 2020. Detection of 2019 novel coronavirus (2019-nCoV) by real-time RT-PCR. Euro Surveill 25. doi: 10.2807/1560 7917.ES.2020.25.3.2000045.[12]
