# Supplementary material for: A case report describing the immune response of an infant with congenital heart disease and severe COVID-19
Source: Commun Med (Lond). 2021 Nov 15;1:47. doi: 10.1038/s43856-021-00047-7 (PMC9053208; doi:10.1038/s43856-021-00047-7)
Supplement: Supplementary file 2 — Description of Additional Supplementary Files [file 43856_2021_47_MOESM2_ESM.pdf]

Description of Additional Supplementary Files

File Name: Supplementary Data 1

Description: Excel table that includes all source data used in this manuscript
