# Supplementary material for: A case report describing the immune response of an infant with congenital heart disease and severe COVID-19
Source: Commun Med (Lond). 2021 Nov 15;1:47. doi: 10.1038/s43856-021-00047-7 (PMC9053208; doi:10.1038/s43856-021-00047-7)
Supplement: Supplementary file 4 — Reporting Summary [file 43856_2021_47_MOESM4_ESM.pdf]

nature research

Corresponding author(s): Paul Licciardi, Daniel Pellicci

Last updated by author(s): Aug 22, 2021

Reporting Summary

Nature Research wishes to improve the reproducibility of the work that we publish. This form provides structure for consistency and transparency in reporting. For further information on Nature Research policies, see our Editorial Policies and the Editorial Policy Checklist.

Statistics

For all statistical analyses, confirm that the following items are present in the figure legend, table legend, main text, or Methods section.

n/a	Confirmed	
☐	☒	The exact sample size (*n*) for each experimental group/condition, given as a discrete number and unit of measurement
☒	☐	A statement on whether measurements were taken from distinct samples or whether the same sample was measured repeatedly
☒	☐	The statistical test(s) used AND whether they are one- or two-sided<br>*Only common tests should be described solely by name; describe more complex techniques in the Methods section.*
☒	☐	A description of all covariates tested
☒	☐	A description of any assumptions or corrections, such as tests of normality and adjustment for multiple comparisons
☒	☐	A full description of the statistical parameters including central tendency (e.g. means) or other basic estimates (e.g. regression coefficient) AND variation (e.g. standard deviation) or associated estimates of uncertainty (e.g. confidence intervals)
☒	☐	For null hypothesis testing, the test statistic (e.g. *F*, *t*, *r*) with confidence intervals, effect sizes, degrees of freedom and *P* value noted<br>*Give P values as exact values whenever suitable.*
☒	☐	For Bayesian analysis, information on the choice of priors and Markov chain Monte Carlo settings
☒	☐	For hierarchical and complex designs, identification of the appropriate level for tests and full reporting of outcomes
☒	☐	Estimates of effect sizes (e.g. Cohen's *d*, Pearson's *r*), indicating how they were calculated

Our web collection on statistics for biologists contains articles on many of the points above.

Software and code

Policy information about availability of computer code

Data collection	REDCap V10.1.2, Microsoft Excel 2009 Office 365, BD FACS DIVA V9.0, Cytek Aurora software SpectroFlo.
Data analysis	Flowjo V10, tSNE, UMAP and FLOWSOM plugins within Flowjo V10, Prism V8 and the Morpheus heatmap tool: https://software.broadinstitute.org/morpheus/. Biorad bioplex analyser for cytokine analyses.

For manuscripts utilizing custom algorithms or software that are central to the research but not yet described in published literature, software must be made available to editors and reviewers. We strongly encourage code deposition in a community repository (e.g. GitHub). See the Nature Research guidelines for submitting code & software for further information.

Data

Policy information about availability of data

All manuscripts must include a data availability statement. This statement should provide the following information, where applicable:

- Accession codes, unique identifiers, or web links for publicly available datasets
- A list of figures that have associated raw data
- A description of any restrictions on data availability

The source data underlying all figures and supplemental data will be provided as a Source Data file

Field-specific reporting

Please select the one below that is the best fit for your research. If you are not sure, read the appropriate sections before making your selection.

☒ Life sciences ☐ Behavioural & social sciences ☐ Ecological, evolutionary & environmental sciences

For a reference copy of the document with all sections, see nature.com/documents/nr-reporting-summary-flat.pdf

Life sciences study design

All studies must disclose on these points even when the disclosure is negative.

Sample size	The case study consists of a 5-month old infant (male), with samples collected at five time points. An aged matched control was included for data in Figures 2 and 3 as well as Supplementary Figure 4.
Data exclusions	No data were excluded from the analysis
Replication	Where biological sample volume permitted, each test was replicated at least once to verify all findings. All attempts at replication were successful.
Randomization	This was a case study selected based on their COVID-19 severity (hospitalisation) . No randomization for participants was done.
Blinding	Investigators were blinded to disease outcome during experimentation and data analysis.

Reporting for specific materials, systems and methods

We require information from authors about some types of materials, experimental systems and methods used in many studies. Here, indicate whether each material, system or method listed is relevant to your study. If you are not sure if a list item applies to your research, read the appropriate section before selecting a response.

Materials & experimental systems

n/a	Involved in the study	
☐	☒	Antibodies
☒	☐	Eukaryotic cell lines
☒	☐	Palaeontology and archaeology
☒	☐	Animals and other organisms
☐	☒	Human research participants
☒	☐	Clinical data
☒	☐	Dual use research of concern

Methods

n/a	Involved in the study	
☒	☐	ChIP-seq
☐	☒	Flow cytometry
☒	☐	MRI-based neuroimaging

Antibodies

Antibodies used

Surface Marker Fluorophore Clone Final Dilution
Whole blood panel
CD14 BV786 MSE2 1:50
CD11b BUV805 ICRF44 1:100
CD45 BV711 HI30 1:100
CD56 BUV737 NCAM16.2 1:100
CD11c PE-Cy7 B-ly6 1:100
CD63 A647 H5C6 1:100
CD4 A700 RPA-T4 1:100
CD3 B8515 VCHTI 1:100
PD1 B8700 EH12.1 1:100
CD15 PE-CF594 W603 1:200
HLADR VSOO G46-6 1:200
CD19 BV605 5J25CI 1:200
CD8 BV650 RPA-T8 1:200
CD16 BUV395 3G8 1:400

PBMC panel
CD25 PE M-A251 1:25
CD127 V450 HIL7RM21 1:50
CD3 APCH7 SK7 1:50
CD14 BV786 MSE2 1:50
CD45RA BV711 HI100 1:100

HLADR BB515 G46-6 1:100
CD56 BUV737 NCAM16.2 1:100
CD11c PE-Cy7 B-ly6 1:100
CD4 A700 RPA-T4 1:100
PD1 BB700 EH12.I 1:100
CCR7 PE-CF594 150503 1:200
CD19 BV605 SJ25Cl 1:200
CD8 BV650 RPA-T8 1:200
CD16 BUV395 3G8 1:400

Cytek Aurora antibodies:
CD3 BUV395 UCHT1 1:100
CD4 BV510 SK3 1:200
CD8 BV805 SK1 1:400
CD25 PE-CF594 M-A251 1:100
CD45RA PerCP/Cy5.5 M1100 1:200
CD69 BV650 FN50 1:200
CD127 APCR700 HIL-7R-M21 1:100
CD161 PEvio770 191B8 1:200
CXCR3 APC IC6/CXCR3 1:50
CCR4 BV605 L29IH4 1:200
CCR6 BV421 11A9 1:100
CCR7 BV785 G043H7 1:100
gdTCR FITC 11F2 1:50
Vd2 PE B6 1:400
Va7.2 BV711 3C10 1:200
Zombie NIR NIR 1:800

Validation

Each lot of conjugated antibody is quality control tested by flow cytometry analysis of stained cells using the appropriate positive and negative cell staining. All antibodies are mouse anti-human, and were tested on human cells.

Human research participants

Policy information about studies involving human research participants

Population characteristics

One 5-month old male hospitalised with severe COVID-19 disease. An age-matched male control was also used.

Recruitment

This patient was recruited into this study at the Royal Children's Hospital based on positive COVID-19 diagnosis.

Ethics oversight

This project received ethical approval from The Royal Children's Hospital Melbourne Human Research Ethics Committee (HREC): HREC 63103

Note that full information on the approval of the study protocol must also be provided in the manuscript.

Flow Cytometry

Plots

Confirm that:

☒ The axis labels state the marker and fluorochrome used (e.g. CD4-FITC).

☒ The axis scales are clearly visible. Include numbers along axes only for bottom left plot of group (a 'group' is an analysis of identical markers).

☒ All plots are contour plots with outliers or pseudocolor plots.

☒ A numerical value for number of cells or percentage (with statistics) is provided.

Methodology

Sample preparation

Whole blood:
Blood was collected in EDTA tubes and 100 µL of whole blood was aliquoted for flow cytometry analysis. Whole blood was lysed with 1 mL of red cell lysis buffer for 10 minutes at room temperature. Cells were washed with 1 mL PBS and centrifuged at 350 x g for 5 minutes. Following two more washes, cells were resuspended in PBS for viability staining using near infra-red viability dye according to manufacturers instructions. The viability dye reaction was stopped by the addition of FACS buffer (2% heat-inactivated FCS in 2 mM EDTA) and cells were centrifuged at 350 x g for 5 minutes. Cells were then resuspended in 23 human FC-block according to manufacturers instructions for 5 minutes at room temperature. The whole blood cocktail (refer Supplementary Table S2) made up at 2X concentration were added 1:1 with the cells and incubated for 30 minutes on ice. Following staining, cells were washed with 2 mL FACS buffer and centrifuged at 350 x g for 5 minutes. Cells were 1 then resuspended in 2% PFA for a 20 minute fixation on ice, washed, and resuspended in 150µL FACS buffer for acquisition using the BD LSR X-20 Fortessa and BD FACS DIVA V 9.0 software. For all flow cytometry experiments, compensation was done at the time of sample acquisition using compensation beads.

PBMCs:
Cryopreserved peripheral blood mononuclear cells (PBMCs) were thawed at 37°C then washed 9 with 10mL R10 media

(RPMI-1640 medium supplemented with 10% fetal bovine serum, 200nM L-glutamine, 1000IU penicillin-streptomycin) and centrifuged at 400 x g for 5 minutes. PBMCs were washed with 5mL PBS and centrifuged at 400 x g for 5 minutes then blocked (50µl of 1% human FC-block and 10% normal rat serum in PBS) for 15 minutes on ice. PBMCs were washed with 1mL FACS buffer and centrifuged at 400 x g for 5 mins then stained with 50µl PBMC cocktail (refer Supplementary Table S3) for 20 minutes on ice. PBMCs were washed then resuspended in 4% PFA for a 10 minute fixation on ice, washed, and resuspended in 100µl FACS buffer for acquisition using the Cytek Aurora. Compensation was performed at the time of acquisition using compensation beads.

Instrument

BD Fortessa X-20 and Cytek Aurora

Software

Data collection: DIVA Data analysis: FlowJo V10

Cell population abundance

No cell populations were sorted.

Gating strategy

Within the PBMC fraction, B cells were selected based on CD19 expression, and the total Tcell fraction based on CD3 expression. CD4 and CD8 T cells, and their naive, effector, memory and regulatory (Treg) subsets were also quantified. HLADR and PD1+ T cells were investigated. CD3-CD19- cells were classified into NK cells (CD56+) and innate cells {HLA-DR+). Within the innate cell fraction, CD14+ monocytes and CD11c+ DCs were identified. Monocyte and NK cell subsets were identified based on CD16 expression. Low density neutrophils were observed in the PBMC fraction at day 88 only, characterised by a high SSC profile, CD16, CD14 and CD11c expression. For whole blood, granulocytes were selected within CD45+ leukocytes based on their SSC profile and CD15 expression. Neutrophils were CD15+CD16+ and eosinophils were CD15+CD16-. Please refer to Supplementary Figure 2.
PBMC gating strategy. T-cells were identified by CD3+ expression on live single lymphocytes. T-cells were further categorised into CD4+, CD8+, γδTCR+Vδ2+ and CD161+Vα7.2+ (MAIT cells). From the CD4+ T-cells we further identified their subsets. CXCR3+ cells were considered Th1, CXCR3-CCR4+CCR6- cells were considered Th2, CXCR3-CCR4+CCR6+CD161+ cells were considered Th17 and CD25hiCD127lo cells were considered Tregs. CCR7 and CD45RA were used to discriminate CD4+ and CD8+ cells into their memory subsets (Central memory (CM), effector memory (EM), naïve (N) and effector (E)). To determine activation status, CD69 was gated from all T-cell subsets, CD4+, CD8+, γδTCR+Vδ2+ and MAIT cells. Please refer to Supplementary Figure 3.

☒ Tick this box to confirm that a figure exemplifying the gating strategy is provided in the Supplementary Information.
